# Polygenic risk score predicting susceptibility and outcome of benign prostatic hyperplasia in the Han Chinese

**DOI:** 10.1186/s40246-024-00619-3

**Published:** 2024-05-22

**Authors:** Sheng-Chun Hung, Li-Wen Chang, Tzu-Hung Hsiao, Guan-Cheng Lin, Shian-Shiang Wang, Jian-Ri Li, I-Chieh Chen

**Affiliations:** 1 Division of Urology, Department of Surgery, Taichung Veterans General Hospital, Taichung, Taiwan; 2 Department of Post-Baccalaureate Medicine, College of Medicine, National Chung Hsing University, Taichung, Taiwan; 3 Institute of Medicine, Chung Shan Medical University, Taichung, Taiwan; 4 Department of Medical Research, Taichung Veterans General Hospital, Taichung, Taiwan; 5 Department of Public Health, Fu Jen Catholic University, New Taipei City, Taiwan; 6 Institute of Genomics and Bioinformatics, National Chung Hsing University, Taichung, Taiwan; 7 Department of Applied Chemistry, National Chi Nan University, Nantou, Taiwan; 8 Department of Medicine and Nursing, Hungkuang University, Taichung, Taiwan

**Keywords:** Benign prostatic hyperplasia, Polygenic risk score, Prostate-specific antigen level, 5-α-reductase inhibitor, Incidence, Prognosis

## Abstract

**Background:**

Given the high prevalence of BPH among elderly men, pinpointing those at elevated risk can aid in early intervention and effective management. This study aimed to explore that polygenic risk score (PRS) is effective in predicting benign prostatic hyperplasia (BPH) incidence, prognosis and risk of operation in Han Chinese.

**Methods:**

A retrospective cohort study included 12,474 male participants (6,237 with BPH and 6,237 non-BPH controls) from the Taiwan Precision Medicine Initiative (TPMI). Genotyping was performed using the Affymetrix Genome-Wide TWB 2.0 SNP Array. PRS was calculated using PGS001865, comprising 1,712 single nucleotide polymorphisms. Logistic regression models assessed the association between PRS and BPH incidence, adjusting for age and prostate-specific antigen (PSA) levels. The study also examined the relationship between PSA, prostate volume, and response to 5-α-reductase inhibitor (5ARI) treatment, as well as the association between PRS and the risk of TURP.

**Results:**

Individuals in the highest PRS quartile (Q4) had a significantly higher risk of BPH compared to the lowest quartile (Q1) (OR = 1.51, 95% CI = 1.274–1.783, *p* < 0.0001), after adjusting for PSA level. The Q4 group exhibited larger prostate volumes and a smaller volume reduction after 5ARI treatment. The Q1 group had a lower cumulative TURP probability at 3, 5, and 10 years compared to the Q4 group. PRS Q4 was an independent risk factor for TURP.

**Conclusions:**

In this Han Chinese cohort, higher PRS was associated with an increased susceptibility to BPH, larger prostate volumes, poorer response to 5ARI treatment, and a higher risk of TURP. Larger prospective studies with longer follow-up are warranted to further validate these findings.

**Supplementary Information:**

The online version contains supplementary material available at 10.1186/s40246-024-00619-3.

## Key points

**Question** What is the role of polygenic risk score (PRS) in predicting the incidence and treatment outcome of benign prostatic hyperplasia (BPH) in Han Chinese?

**Findings** In this hospital-based genome-wide association study (GWAS) involving 23,407 male participants, it was discovered that Han Chinese individuals with a higher polygenic risk score (PRS) showed an increased susceptibility to benign prostatic hyperplasia (BPH). Furthermore, patients with elevated PRS exhibited higher levels of prostate-specific antigen (PSA), larger prostate volume, a diminished response to 5-alpha-reductase inhibitor (5ARI) treatment, and a heightened risk of undergoing transurethral resection of the prostate (TURP).

**Meaning** These findings indicate that PRS can be a valuable predictive tool for assessing the risk of BPH incidence and treatment outcomes in male Han Chinese individuals. However, further large-scale studies with longer follow-up periods are necessary to validate these findings and establish the clinical utility of PRS in BPH management.

**Supplementary Information:**

The online version contains supplementary material available at 10.1186/s40246-024-00619-3.

## Introduction

Benign prostatic hyperplasia (BPH) is a common condition in elderly men characterized by an enlargement of the prostate gland, leading to low urinary tract symptoms such as frequency, urgency, intermittency, weak stream, strain, and incomplete emptying of the bladder [1]. Additionally, residual urine or increased bladder pressure would cause urinary tract infection, bladder stone, obstructive nephropathy or kidney injury. BPH affects approximately 50% of men over the age of 50 and 90% of men over the age of 80, making it a significant health concern in aging populations worldwide [2].

The exact cause of BPH is not fully understood, but it is believed to be a complex interplay between genetic and environmental factors [3–5]. Genetic factors play a crucial role in the development of BPH, with several genes and single nucleotide polymorphisms (SNPs) identified to be associated with an increased risk of BPH [6–8]. These genetic variations may influence the growth of prostate glandular cells, androgen and androgen receptors expression, epithelial-stromal interactions, inflammation, and other biological processes involved in the pathogenesis of BPH [9]. To date, it is few genome-wide significant risk variants of BPH were reported in the Asia ancestry [10, 11]. The established BPH Polygenic Risk Score (PRS) largely relies on the independent risk variants that have been previously identified in individuals of European ancestry [12, 13], and study population almost was conducted in a large cohort from the UK Biobank [14].

The recommended initial treatment for male patients with BPH or low urinary tract symptoms is alpha-1 adrenoceptor antagonists [15]. If patients continue to experience voiding difficulties despite alpha-1 adrenoceptor antagonists, 5-α-reductase inhibitor (5ARI) can be used to achieve a prostate volume reduction of 18–28% and improve low urinary tract symptoms by 15–30% through the reduction of dihydrotestosterone [16–18]. In cases where medical treatment fails, patients may undergo surgical intervention such as monopolar transurethral resection of the prostate (TURP), bipolar TURP or laser TURP, or they may receive a combination of pharmacological and surgical treatments to relieve outlet obstruction [19].

PRS is a recently developed approach that integrates multiple genetic variants to estimate an individual’s genetic predisposition to a specific disease or condition [20]. The PRS is typically calculated by assigning weights to each SNPs based on their estimated influence, and then summing these weighted SNPs together. This allows researchers to evaluate the cumulative contribution of these genetic variants to disease prevalence and incidence in a population [20]. PRS has been widely used in the field of genetics to predict the risk of various diseases, such as cancer, cardiovascular disease, and diabetes [21–23]. PRS has also been utilized to evaluate the genetic risk of developing BPH and identify individuals who may be at a higher risk of developing severe symptoms. For instance, a PRS composed of 23 genetic variants has been shown to be associated with elevated levels of prostate-specific antigen (PSA) and an increased likelihood of BPH development [24].

In this study, we aim to explore the association between BPH and PRS, specifically examining risk of BPH development, prostate volume in patients with BPH, response to 5ARI treatment, and the risk of TURP in the Asian population. This investigation will be conducted using genome-wide association analysis (GWAS) and data obtained from the Taiwan Precision Medicine Initiative (TPMI).

## Patients and methods

### Study population

Between June 2019 and May 2021, Taichung Veterans General Hospital (TCVGH), a tertiary medical center, conducted patient recruitment for the TPMI project, a nationwide genetic research endeavor led by Academia Sinica in Taiwan. The study received approval from the ethics committee of TCVGH Institutional Review Board (IRB No. CE23119A), and informed consent was obtained from all participants. Male participants were exclusively included in the analysis and patients with confirmed prostate cancer were excluded. Clinical parameters were sourced from the dataset and electronic medical records of TCVGH, utilizing de-identification methods. This study follows the PRS-RS guidelines (Appendix 1).

### Genotyping and imputation

The blood samples were genotyped using the Axiom Genome-Wide TWB 2.0 Array Plate (Affymetrix, Santa Clara, CA, USA), which was specifically designed for the Han Chinese population in Taiwan and contains 714,431 SNPs [25]. Quality control and analysis were performed using Affymetrix Power Tools to eliminate markers that did not pass Hardy-Weinberg equilibrium tests (*P* < 1.0 × 10^–5^), had a minor allele frequency less than 0.05, or had a genotype missing rate exceeding 5%, as previously described [26]. All participants in the TPMI cohort underwent genotyping, resulting in data for approximately 700,000 genetic variants. Genetic variants were aligned to the human genome reference GRCh38. In our study, genotype imputation was primarily conducted to ensure the completeness and consistency of the dataset for subsequent analyses. Genotype imputation is a method used to estimate missing or unobserved genotype data in order to fill gaps in the dataset. Genotype imputation was conducted across the autosomal chromosomes utilizing the Michigan Imputation Server, employing the ‘minimac4’ algorithm as detailed in previous literature [27]. The genotype data, aligned with the DNA strands, were uploaded to the server. The imputation process was carried out with the aid of the 1000 Genomes Phase 3 (Version 5) reference panel [28], resulting in a final set of 4.9 million sties and 2,504 samples. After excluding variants that did not meet quality control criteria, an additional approximately 3 million genetic variants were imputed. Variant IDs were assigned based on the Genome Reference Consortium Human Build 37 (GRCh37) reference genome [29]. All biallelic variants meeting the INFO score threshold of ≥ 0.3 were included in the report. After aligning the genetic variants to the human genome reference GRCh38, and following the genotype imputation process and quality control criteria, a total of 1,692 variants from the PGS001865 PRS [13] were included in the analysis for the Han Chinese population (Supplemental Table 1).

### Linkage disequilibrium

Linkage disequilibrium (LD) is the non-random association of alleles at different genetic loci in a population. When two genetic variants, like single nucleotide polymorphisms (SNPs), exhibit strong LD, they tend to be inherited together more frequently than by chance. LD can arise from physical proximity on a chromosome or other evolutionary factors. LD pruning and clumping, performed here using plink version 2.0 (PLINK 2) [30, 31], help select independent and informative SNPs for PRS development, avoiding multicollinearity issues in statistical modeling.

### Polygenic risk score analysis

We computed the PRS using the ‘score’ function of PLINK 2, which combines the weighted effects of genetic variants based on their effect size [30, 32]. When the effect and non-effect allele are inverted between datasets then this can be resolved automatically by PLINK 2. In this study, we utilized the PGS001865 PRS, which was derived from a discovery analysis of 1,712 variants associated with BPH identified from a Trans-ancestry GWAS for 245 curated traits across nine ancestry groups from the United Kingdom (UK) Biobank [13]. We obtained the list of SNPs and their corresponding effect sizes from the Polygenic score (PGS) catalog [33]. The approach we utilized is to weight each allele dosage by its effect size, as described previously [34]. The equation is a standard formula used to calculate a weighted PRS:

1 $$\begin{array}{c}\backslash textPR{\text{S}}_{\text{j}}={\sum }_{\text{i}}^{\text{N}}{{\upbeta }}_{\text{i}}\times \backslash textdosag{\text{e}}_{\text{i}\text{j}}\#\left(1\right)\end{array}$$

Equation (1) calculates a weighted PRS for individual j. N represents the total number of SNPs from GWAS used in the PRS calculation. Furthermore, ${{\upbeta }}_{\text{i}}$ signifies the effect size of the effect allele of SNP_i_, while dosage_ij_ is the number of copies of the effect allele in the genotype of individual j.

Finally, this PRS was constructed using a panel of 1,692 SNPs from the PGS001865 [13], exhibited predictive performance in our cohort, with an area under the curve (AUC) of 0.526 for BPH. Furthermore, the C-index indicates predictive accuracy for PGS001865 (C-index: 0.546, 95% CI, 0.504–0.666).

### Clinical parameters and outcome evaluation

BPH cases were identified using the International Classification of Diseases, Ninth Revision Clinical Modification (ICD-9-CM) code 600.x with available medical records (Supplemental Table 2). Initially, we analyzed the incidence of BPH among male participants. Subsequently, the genetic profile of the participants was linked to clinical parameters, including age of diagnosis, PSA levels, and medical history. Prostate volume was assessed using transrectal sonography, computed tomography (CT), magnetic resonance imaging (MRI), or transabdominal songraphy. The response to 5-α-reductase inhibitor (5ARI) medication was determined as the percentage reduction in prostate volume after treatment. Outcome evaluation was based on the incidence of patients who underwent monopolar, bipolar, or laser TURP. The association between PRS and BPH development was examined using logistic regression analysis.

### Statistical analysis

In order to assess the association between PRS and the outcome of interest, the PRS values were classified into four groups based on quartiles, denoted as Q1 (0–25%), Q2 (26–50%), Q3 (51–75%), and Q4 (76–100%). Descriptive statistics for continuous variables were presented as mean ± standard deviation (SD), and differences among groups were analyzed using Student’s t-test. Categorical variables were presented as number (percent), and group differences were evaluated using Chi-square test. To further examine the relationship between PRS and the outcome, univariable logistic regression analysis was conducted to calculate odds ratio (OR) and 95% confidence interval (95% CI). The risk of BPH surgery was explored using the univariable and multivariable Cox proportional hazards model, and for the time variable, we used the patient’s age at the time of the surgery or last follow-up (as of December 31, 2022). The hazard ratio (HR) and corresponding *p*-value were reported. All statistical analyses were performed using IBM SPSS statistical software for Windows, version 25.0 (IBM Corp., Armonk, NY, United States).

## Results

### Baseline characteristics of the study participants

**Fig. 1:**
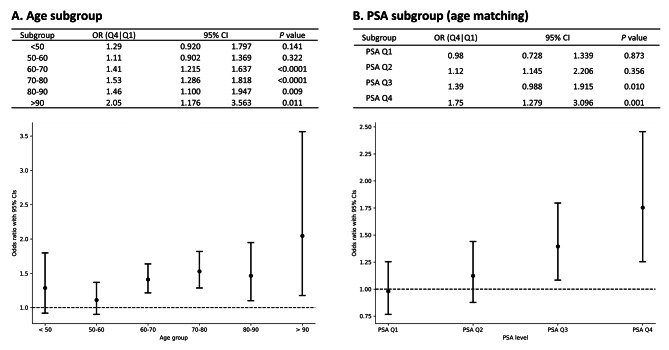
Risk for incident BPH in study subject (*N* = 12,474). **(A)** Age subgroup. The x-axis indicates the age of an individual; **(B)** PSA subgroup by age matching. The x-axis indicates the four quartiles of PSA. The y-axis is the odds ratio (OR) for BPH.

Of the 57,257 participants from the TPMI project at TCVGH, a total of 23,407 male participants were included in the analysis. The BPH cohort consisted of 6,237 male cases, matched 1:1 to 6,237 male controls based on age using nearest neighbor matching (Supplementary Fig. 1). The incidence of BPH increased from 45.6% in the lowest polygenic risk score (PRS) quartile (Q1) to 55.0% in the highest quartile (Q4) (Supplemental Table 3). As shown in Table 1, the PRS score was associated with an increased risk of BPH, with the risk being significantly higher in Q4 compared to Q1 (OR = 1.51, 95% CI = 1.274–1.783, *p* < 0.0001), after adjusting for prostate-specific antigen (PSA) level. Subgroup analysis by age group showed the risk was higher in Q4 versus Q1, particularly in the 60–70 years (OR = 1.41, 95% CI = 1.215–1.637, *p* < 0.0001), 70–80 years (OR = 1.53, 95% CI = 1.286–1.818, *p* < 0.0001), and over 90 years (OR = 2.05, 95% CI = 1.176–3.563, *p* = 0.011) age groups (see Figure 1A).

### Association between PRS and risk of BPH

Table 2 presents the characteristics of patients with BPH. No significant differences were observed in terms of diagnosis age, smoking, 5ARI usage time, hypertension, and diabetes mellitus among the different quartiles of PRS groups. However, prostate-specific antigen (PSA) levels increased significantly across the PRS quartiles, from a median of 1.09 ng/mL (IQR 0.63–2.28) in Q1 to 1.78 ng/mL (IQR 0.95–3.87) in Q4 (*p* < 0.001). After stratification by PSA quartiles, the incidence of BPH remained elevated in the highest PRS quartile (Q4) compared to the lowest (Q1) within the highest PSA quartile (Q4) group (OR = 1.75, 95% CI = 1.279–3.096, *p* = 0.001) and the third PSA quartile (Q3) group (OR = 1.39, 95% CI = 0.988–1.915, *p* = 0.010) (see Fig. 1B). These findings indicate that the PRS can independently predict the incidence of BPH, regardless of PSA values.

**Table 1:** Association of PRS (PGS001865) and BPH in study population (*N* = 12,474)

Variables	OR	95% CI		*P* value^a^
Age-matched				
Q4|Q1	1.51	1.274	1.783	< 0.0001
Q3|Q1	1.24	1.053	1.463	0.010
Q2|Q1	1.15	0.972	1.349	0.105

^a^ Comparisons of categorical variables were analyzed using the logistic regression adjusted by PSA level

Abbreviation: BPH = benign prostatic hyperplasia, PRS = polygenic risk score, OR = Odd ratio.

**Table 2:** Characteristics of the patients with BPH separated by PRS quartile

Variables		Quartile of polygenic risk score (PGS001865)								*P* value^a^	
		Q1 (*n* = 1377)		Q2 (*n* = 1525)		Q3 (*n* = 1594)		Q4 (*n* = 1741)			
Demography											
	Diagnosis age	63 ± 11		63 ± 11		63 ± 11		63 ± 11		0.46	
	Smoking										
	No	619	45.0	659	43.2	714	44.8	757	43.5		
	Yes	758	55.0	866	56.8	880	55.2	984	56.5	0.69	
	PSA, median (IQR)	1.09 (0.63–2.28)		1.33 (0.70–2.84)		1.55 (0.84–3.50)		1.78 (0.95–3.87)		< 0.001	
	5ARI usage time (day)	439 ± 609		450 ± 681		432 ± 623		435 ± 642		0.98	
Comorbidities											
	Hypertension										
	No	571	41.5	637	41.8	676	42.4	716	41.1		
	Yes	806	58.5	888	58.2	918	57.6	1025	58.9	0.90	
	Diabetes mellitus										
	No	810	58.8	904	59.3	948	59.5	1027	59.0		
	Yes	567	41.2	621	40.7	646	40.5	714	41.0	0.98	

^a^ Comparisons of continuous variables using Student’s t-test and categorical variables were analyzed using the Chi-square test

Abbreviation: BPH = benign prostatic hyperplasia, PSA = prostate specific antigen, IQR = interquartile range, 5ARI = 5-α-reductase inhibitor

The characteristics of this retrospective matched cohort study are presented in Supplemental Table 4. The BPH case group was slightly older than the control group (70 ± 12 vs. 69 ± 11 years, *p* < 0.001), due to age matching using nearest neighbor matching. Compared to controls, the BPH cases had higher rates of smoking (55.9% vs. 48.9%, *p* < 0.001), hypertension (58.3% vs. 50.3%, *p* < 0.001), hyperlipidemia (50.2% vs. 43.9%, *p* < 0.001), and cancer (52.0% vs. 41.6%, *p* < 0.001). Furthermore, in order to elucidate the correlation between prostate volume and the PRS, participants were classified into three categories: BPH with a prostate volume > 40 mL, BPH with a prostate volume < 40 mL, and a control group. Our findings reveal a statistically significant elevation in PRS among individuals with BPH and a prostate volume > 40 mL compared to those with a prostate volume < 40 mL (*p* < 0.001). Similarly, PRS was significantly higher in individuals with BPH and a prostate volume < 40 mL compared to the control group (*p* = 0.016) (Supplementary Fig. 2).

### Association of PRS and clinical parameters in BPH patients

Table 3 demonstrates the outcomes of patients with BPH. Prostate volume was found to increase significantly across the PRS quartiles, from 34.6 ± 19.0 mL in Q1 to 43.1 ± 25.0 mL in Q4 (*p* < 0.001). The response to 5ARI medication, measured as reduction in prostate volume, was inferior in Q4 compared to Q1 (25.0 ± 18.6 mL vs. 29.9 ± 16.6 mL, respectively). The risk of patients undergoing TURP was found to be higher in Q4 (Q1 to Q4: 5.8%, 6.5%, 7.8%, and 10.1%, respectively, *p* < 0.001) (Table 3).

**Table 3:** Outcomes of patients with BPH separated by PRS quartile

Variables	Quartile of polygenic risk score (PGS001865)								*P* value^a^
	Q1 (*n* = 1377)		Q2 (*n* = 1525)		Q3 (*n* = 1594)		Q4 (*n* = 1741)		
Prostate volume (ml)	34.6 ± 19.0		37.3 ± 21.4		41.3 ± 24.2		43.1 ± 25.0		< 0.001
Reduction volume after 5ARI (%)	29.9 ± 16.6		30.4 ± 16.8		26.7 ± 17.4		25.0 ± 18.6		0.011
TURP (n, %)									
No	1297	94.2	1426	93.5	1469	92.2	1565	89.9	
Yes	80	5.8	99	6.5	125	7.8	176	10.1	< 0.001

a Comparisons of continuous variables using Student’s t-test and categorical variables were analyzed using the Chi-square test

Abbreviation: BPH = benign prostatic hyperplasia, 5ARI = 5-α-reductase inhibitor, TURP = transurethral resection of prostate

**Fig. 2:**
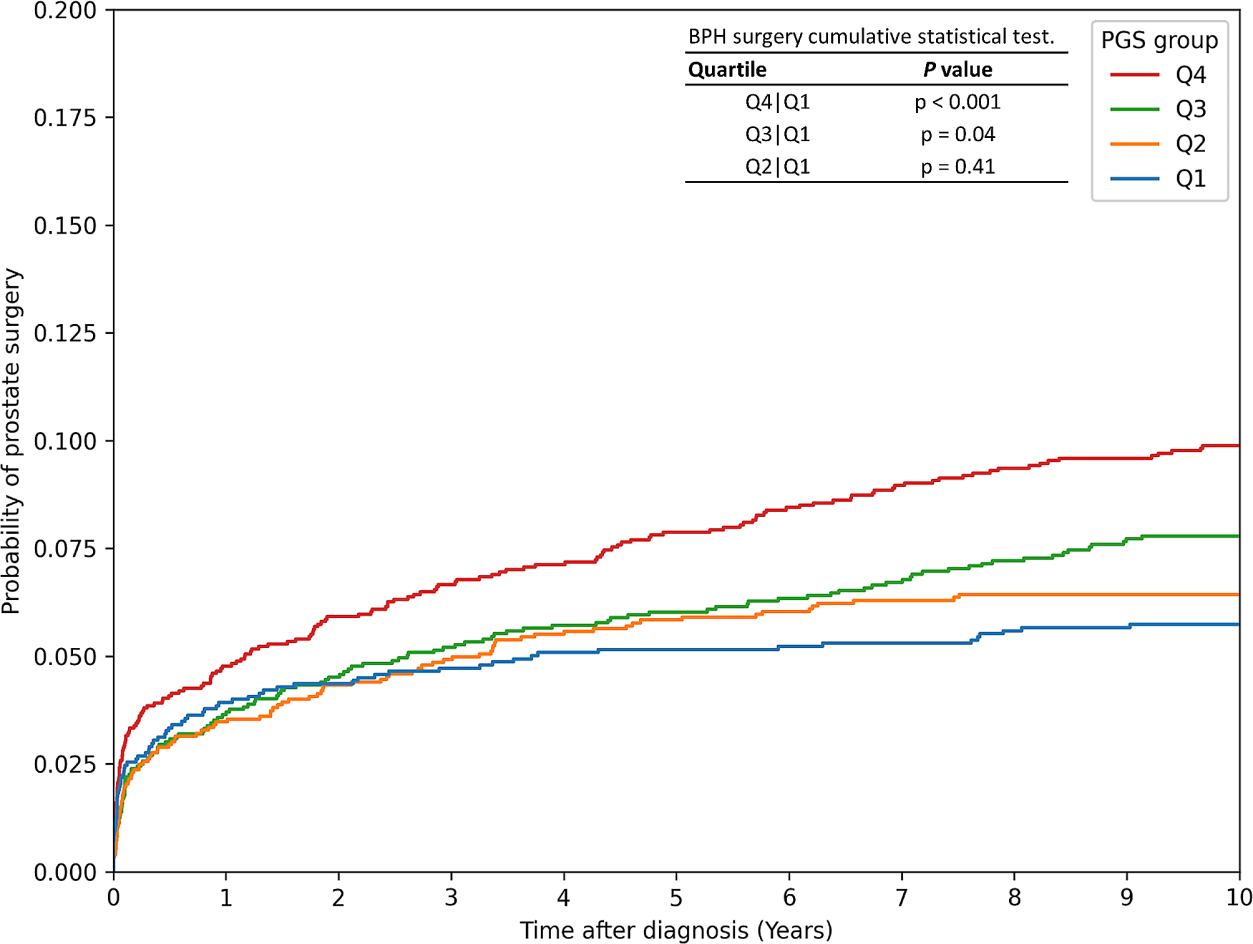
The cumulative incidence of transurethral resection of the prostate (TURP) was compared among the four quartile groups. The results showed a significant difference between Q4 and Q1 (*p* < 0.001). A significant difference was also observed between Q3 and Q1 (*p* = 0.04). However, there was no significant difference between Q2 and Q1 (*p* = 0.41).

Table 4 presents the adjusted TURP probability over time, with significant differences observed starting from the third year. The 3-year TURP probability increased from 4.7% in Q1 to 6.7% in Q4 (*p* = 0.045). The 5-year TURP probability increased from 5.2% in Q1 to 7.9% in Q4 (*p* = 0.009). The 10-year TURP probability increased from 5.7% in Q1 to 9.9% in Q4 (*p* < 0.001). Figure 2 displays the cumulative incidence of TURP, which was significantly higher in Q4 compared to the other quartiles (*p* < 0.001). Table 5 employed uni- and multivariable Cox hazard regression to investigate the risk factors for TURP in BPH. After adjusting for age, prostate volume, and PSA, we found that the highest PRS quartile (Q4) was an independent risk factor, with a hazard ratio (HR) of 1.45 (95% CI = 1.090–1.920, *p* = 0.012) compared to Q1. Additionally, age (HR = 1.010, 95% CI = 1.001–1.019, *p* = 0.028), prostate volume (HR = 1.013, 95% CI = 1.011–1.016, *p* < 0.001), and PSA (HR = 1.017, 95% CI = 1.009–1.025, *p* < 0.001) were identified as other independent risk factors for TURP.

**Table 4:** TURP probability for BPH patients separated by PRS quartile

Variables	Quartile of polygenic risk score (PGS001865)				*P* value^a^
	Q1	Q2	Q3	Q4	
Time (year)					
1	0.039	0.035	0.037	0.048	0.188
3	0.047	0.050	0.052	0.067	0.045
5	0.052	0.059	0.060	0.079	0.009
10	0.057	0.064	0.078	0.099	< 0.001

^a^ categorical variables were expressed as percent and analyzed using the Chi-square test

Abbreviation: BPH = benign prostatic hyperplasia, TURP = transurethral resection of prostate

**Table 5:** TURP risk in BPH patients determined by genetic and non-genetic risk factors

Variables	Univariable model				Multivariable model		
	HR	95% CI	*P* value^a^		HR	95% CI	*P* value^a^
Age	1.025	1.017–1.033	< 0.001		1.010	1.001–1.019	0.028
Quartile							
PRS Q1	1	-	-		1	-	-
PRS Q2	0.810	0.650–1.010	0.059		1.080	0.790–1.480	0.615
PRS Q3	1.020	0.830–1.240	0.889		1.110	0.820–1.500	0.505
PRS Q4	1.500	1.250–1.810	< 0.001		1.450	1.090–1.920	0.012
Prostate volume (ml)	1.014	1.012–1.016	< 0.001		1.013	1.011–1.016	< 0.001
PSA	1.009	1.006–1.013	< 0.001		1.017	1.009–1.025	< 0.001

a Cox proportional hazard regression

Abbreviation: BPH = benign prostatic hyperplasia, HR = hazard ratio, PRS = polygenic risk score, PSA = prostate specific antigen

## Discussion

Our study represents the first to demonstrate a significant association between PRS and BPH within the Han Chinese population. Through the analysis of data gathered from the TCVGH-TPMI cohort, we discovered that PRS serves as a reliable predictor of BPH incidence, as well as other clinical parameters, including prostate volume, response to 5ARI medications, and the future risk of requiring TURP.

As BPH is a prevalent condition among elderly men, identifying individuals at a higher risk of developing it can assist with early intervention and management. The heritability of BPH and male lower urinary tract symptoms in twins has been estimated to range from 20 to 83% across various cohorts, indicating that genetic factors play a significant role in the development of BPH [5, 35, 36]. The first study to report concordance rates for benign prostatic hyperplasia (BPH) among twins who served in the United States military, conducted by the medical follow-up agency, found a rate of 25.7% for monozygotic twins and only 8.5% for dizygotic twins, indicating a significant hereditary influence [35]. Another population-based study compared eighty-three pairs of monozygotic twins with eighty-three pairs of dizygotic twins and concluded that heritability accounts for 82.6% of the variability in symptom scores in men over 50 years of age [36]. However, a large-scale population-based study involving 1,002 pairs of monozygotic twins and 580 pairs of dizygotic twins found that heritability estimates varied, ranging from 21% for nocturia to 40% for straining, with moderate heritability (34–36%) observed for urinary frequency and urgency [5]. Despite variations in heritability estimates, evidence suggested BPH is still influenced by genetic factors.

A multitude of genes have been implicated in the development of BPH. Among these genes, steroid 5α-reductase 1 (*SRD5A1*) and steroid 5α-reductase 2 (*SRD5A2*) encode 5α-reductase type 1 and type 2, respectively, which are involved in testosterone metabolism and have been associated with an increased risk of prostatic hyperplasia [37, 38]. *SRD5A2* is abundantly expressed in the male reproductive system, where it catalyzes the conversion of testosterone to dihydrotestosterone (DHT) [39]. A decrease in the expression of *SRD5A2* has been associated with underdeveloped and atypical genitalia, while an increase in its expression has been linked to excessive DHT production and an increased risk of BPH [40, 41]. 5ARIs, such as finasteride and dutasteride, primarily target *SRD5A2* and reduce the levels of DHT, thereby indicating their effectiveness in treating BPH [41].

The androgen receptor (*AR*) signaling pathway has been established as a key contributor to BPH development. Increased *AR* expression in stromal cells can modify the interaction between epithelial and stromal cells, making the prostate more sensitive to androgen and resulting in hyperplastic growth [42]. Blocking or decreasing *AR* signaling pathway expression could decrease prostate volume and relieve lower urinary tract symptoms [42–44]. Specific *AR* gene polymorphisms, such as SNP rs6152 in the first exon of the *AR* gene, have been associated with *AR* expression and the development of androgenetic alopecia and BPH [45]. Genomic studies have suggested that *FOXA1* regulates the expression of *AR* and that deletion of *FOXA1* in the luminal epithelium of adult mice causes prostatic hyperplasia [46, 47]. Additionally, *NFIB* has been found to interact with *FOXA1* as a cofactor in regulating *AR* target gene expression, and loss of *NFIB* induces prostatic hyperplasia in a mouse model [48]. Although the precise mechanism by which these transcription factors contribute to the pathogenesis of BPH remains unclear, various potential genes have been identified to exhibit association with the development of BPH through different etiologies.

As shown in Supplementary Fig. 3, the protein-protein association involved in PRS were obtained through qualitative networks of the STRING database. The protein-protein association network has revealed potential genetic effects linked to BPH. Fibroblast growth factor receptors (FGFRs) participate in various biological processes including differentiation, motility, and proliferation. Overexpression of the FGFR family has been correlated with BPH [49, 50]. CD34, a member of the single-pass transmembrane protein family, plays a key role in angiogenesis and serves as a reliable marker associated with the development of prostate hyperplasia or prostate cancer [51]. CD34 overexpression is specifically associated with the development of stromal nodules in benign prostatic hyperplasia [52]. Telomerase reverse transcriptase (TERT) is responsible for catalyzing the addition of nucleotides in a TTAGGG sequence to the ends of chromosome telomeres, thereby preventing degradation of chromosomal ends following multiple rounds of replication [53]. Assessing telomerase activity and telomere length may prove useful in the diagnosis and prognosis of prostate cancer [54]. SNP of *TERT* also play a role in determining the risk of BPH in the Chinese Han population [55]. Hepatocyte nuclear factor 1 beta (*HNF1B*) belongs to a family of transcription factors crucial for regulating the development of various tissues and organs during embryogenesis. Overexpression of *HNF1B* is associated with tumorigenesis of solid tumors [56]. Expression of *HNF1B* is also associated with the development of BPH or prostate cancer [57]. Based on the aforementioned studies, comparing our experimental results, we can elucidate the association between benign prostatic hyperplasia and genetics.

The PRS, which is based on multiple genetic markers associated with BPH, can provide a comprehensive risk assessment that takes into account the genetic predisposition of an individual [20]. The positive correlation between PRS and BPH incidence suggests that individuals with higher PRS may have a higher risk of developing BPH, and close monitoring and appropriate interventions should be considered in this population.

Gudmundsson et al. provided initial evidence for a positive association between PRS and BPH. Their study utilized data from the Icelandic BPH/LUTS dataset and the UK Biobank dataset, which included a total of 20,621 BPH patients and 280,541 controls of European ancestry. Through genome-wide analysis, the researchers identified 23 variants at 14 loci that were significantly associated with prostate-specific antigen (PSA) and BPH development (*r*_g_ = 0.77, *P* = 2.6 × 10^− 11^) and one standard deviation increase in a polygenic risk score (PRS) for BPH/LUTS increases PSA levels by 12.9% (*P* = 1.6 × 10^− 55^) [24]. Glaser et al. utilized genome-wide data from the UK Biobank to investigate the genetic relationship between BPH and prostate cancer and they found that the two conditions share common inherited genetics (*r*_g_ = 0.16; 95% CI 0.03–0.28, *p* = 0.01) [58]. In addition to the study by Conti et al., which identified 239 risk SNPs for prostate cancer, Glaser et al. identified 15 risk SNPs for BPH development, and 49 of the identified SNPs were significantly associated with the risk of the other disease (*p* < 0.05) [58, 59]. Furthermore, the PRS for BPH was significantly associated with prostate risk (OR = 1.26, 95% CI 1.18–1.36, *p* < 0.001) and PRS for prostate cancer was significantly associated with BPH risk (OR = 1.03, 95% CI 1.02–1.04, *p* < 0.001), respectively [58]. This suggests that PRS could serve as a comprehensive risk assessment tool for predicting the development of both BPH and prostate cancer. In summary, genetics play a significant role in the occurrence of BPH, and PRS can provide valuable insights for disease prediction.

Our research findings demonstrate a positive correlation between PRS and susceptibility to BPH. Notably, our study deviates from previous literature that has predominantly focused on individuals of European ancestry, as we have specifically analyzed the Han Chinese population. Additionally, patients with prostate cancer were excluded from analysis to prevent any potential confounding factors. As a single-center cohort study, our research benefits from ample medical record availability. We leveraged this advantage by measuring prostate volume as an outcome and discovered that genome-wide factors not only correlated with disease diagnosis [60, 61]. To the best of our knowledge, this is the first report of such a correlation in the literature. Furthermore, our findings echo those of Gudmundsson et al., as we also observed an association between PRS and elevated PSA levels [24].

Secondly, our study revealed that a lower PRS score was associated with a more favorable response to 5ARI treatment. 5ARI medications, such as finasteride and dutasteride, work by inhibiting the conversion of testosterone to dihydrotestosterone (DHT), which is a key hormonal factor in the development of BPH [62]. 5ARI is known to inhibit the activity of the enzyme 5α-reductase, leading to a decrease in DHT levels, induction of apoptosis in prostate epithelial cells, and a resulting reduction in prostate size by approximately 18–28% [63, 64]. The main target of 5ARI, SRD5A2, is an integral membrane enzyme involved in steroid metabolism. It catalyzes the conversion of testosterone to DHT [65]. Prior research has linked high expression of *SRD5A2* to increased prostate volume and metabolic syndromes [37]. Additionally, Gu et al. reported 11 tagging SNPs in the *SRD5A1* and *SRD5A2* genes. Among them, rs523349 and rs9332975 at *SRD5A2* were significantly associated with baseline prostate volume, rs166050 at *SRD5A1* was associated with the posttreatment change in total prostate volume by 5ARI [66, 67]. Furthermore, Austin et al. reported that activation of NF-κB and AR variant 7 (*AR-V7*) could result in increased *SRD5A2* expression, leading to in vivo prostate growth resistant to 5ARI treatment [68]. Conclusively, the finding that PRS is associated with the response to 5ARI medications suggests that genetic factors may influence the efficacy of these medications. PRS can potentially be used to identify individuals who are more likely to respond to 5ARI medications and those who may require alternative treatment options.

Finally, this study also reveals that PRS can predict the risk of requiring TURP in the future. TURP is a common surgical intervention for BPH that is indicated when conservative treatments fail to provide adequate symptom relief [69, 70]. The finding that PRS is associated with the risk of requiring TURP suggests that genetic factors may influence the disease progression of BPH and the need for surgical intervention. PRS can potentially be used as a prognostic tool to identify individuals at higher risk of requiring TURP in the future, and appropriate management strategies can be implemented accordingly.

There are several limitations to this study that should be acknowledged. Firstly, the PRS is based on genetic markers that have been identified in previous studies, and the selection of genetic markers may not be comprehensive. Our study was unable to provide a definitive explanation for the specific impact of individual genes on the development of benign prostatic hyperplasia. Further research is needed to identify additional genetic markers that may contribute to the prediction of BPH incidence and related parameters. Secondly, the sample size of this study may be relatively small, and larger studies are needed to validate the findings and establish the clinical utility of PRS in predicting BPH outcomes. Additionally, PGS001865 is trained on European populations, and its poor portability to Han Chinese individuals leads to limited performance within Han Chinese populations. Consequently, some of the currently published PRS models may not directly apply to Han Chinese or other ethnic populations. Therefore, further studies in diverse populations are necessary to assess the generalizability of these findings. Furthermore, the development of PRS models specifically tailored to Han Chinese individuals is warranted in the future. This would not only enhance predictive accuracy but also improve the clinical utility of PRS models in this population.

## Conclusions

In this hospital-based cohort, a higher PRS was associated with the susceptibility to BPH in male Han Chinese. In patients with BPH, a higher PRS was associated higher PSA level, larger prostate volume, inferior response of 5ARI and higher risk of TURP. Age, PSA and prostate volume were also independent risk of TURP. Prospective large-scale study with longer follow-up would be needed to validate our result.

### Electronic supplementary material

Below is the link to the electronic supplementary material.

Supplementary Material 1. Supplementary Fig. 1. Illustrative flow chart of the study design.

Supplementary Material 2. Supplementary Fig. 2. A boxplot illustrating the variations in PRS median in prostate volume > 40 (ml), prostate volume < = 40 (ml), and control group for the study subjects.

Supplementary Material 3

Supplementary Material 4

Supplementary Material 5. Supplementary Fig. 3. Protein-protein association network of gene sets in PGS001865 by STRING enrichment analysis.

Supplementary Material 6

## Data Availability

The original contributions presented in the study are included in the article and further inquiries can be directed to the corresponding author.

## Data sharing statement

All data used in this study are available in this article.
