# Supplementary figures and images for: Polygenic risk score predicting susceptibility and outcome of benign prostatic hyperplasia in the Han Chinese
Source: Hum Genomics. 2024 May 22;18:49. doi: 10.1186/s40246-024-00619-3 (PMC11110300; doi:10.1186/s40246-024-00619-3)

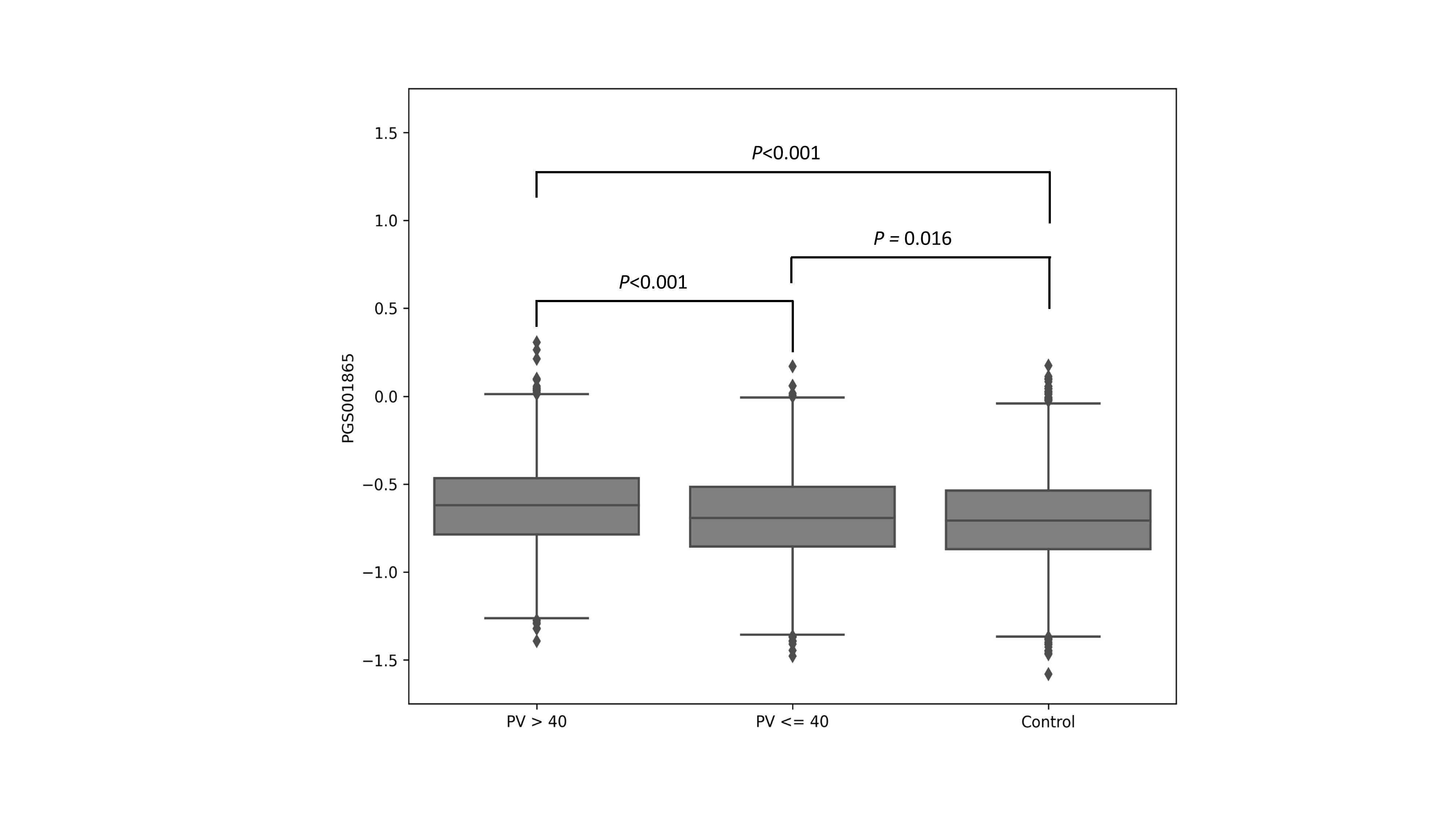

Supplement: Supplementary file 1 — Supplementary Material 1. Supplementary Fig. 1. Illustrative flow chart of the study design. [file 40246_2024_619_MOESM1_ESM.tiff]

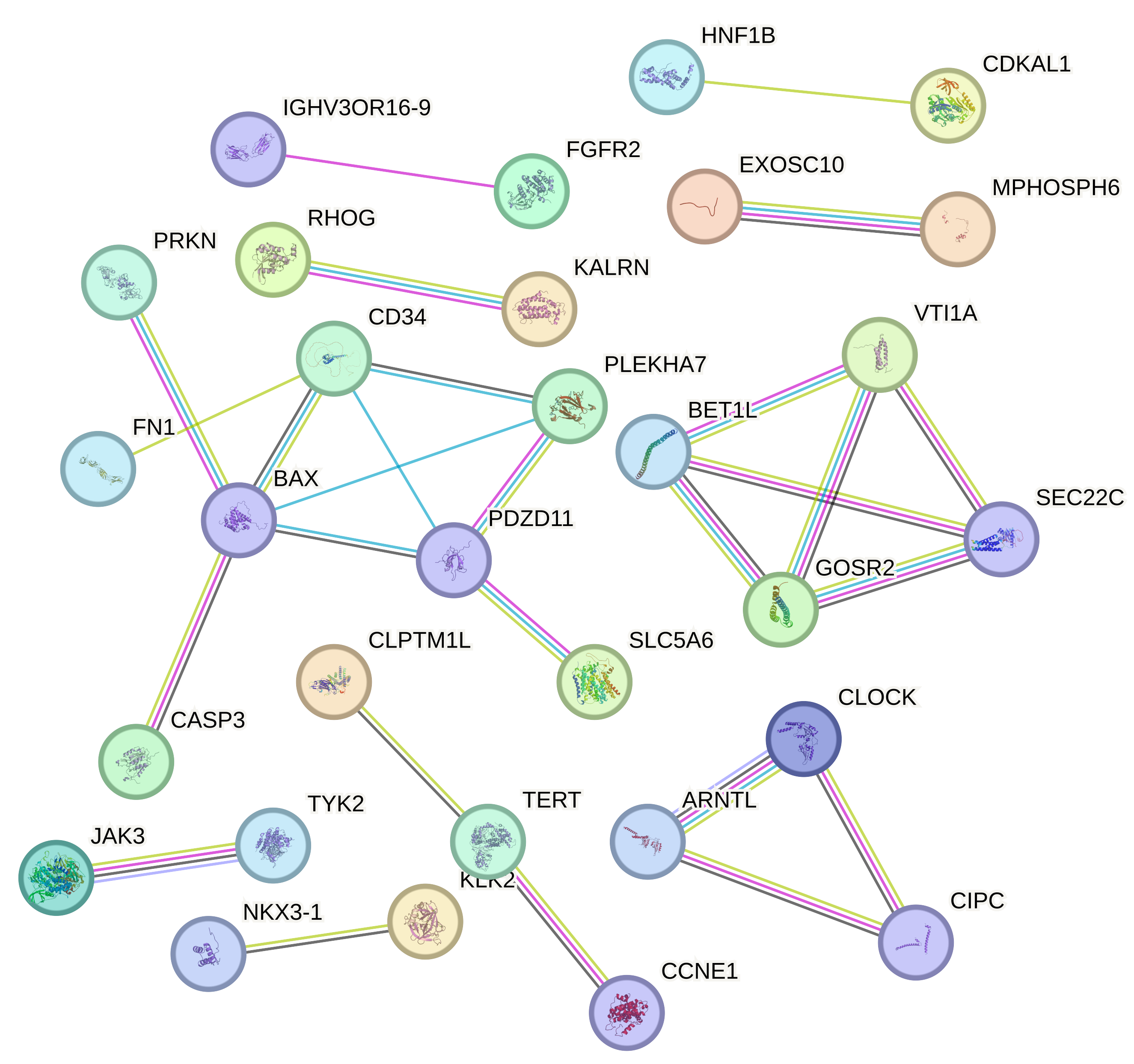

Supplement: Supplementary file 2 — Supplementary Material 2. Supplementary Fig. 2. A boxplot illustrating the variations in PRS median in prostate volume > 40 (ml), prostate volume < = 40 (ml), and control group for the study subjects. [file 40246_2024_619_MOESM2_ESM.tiff]

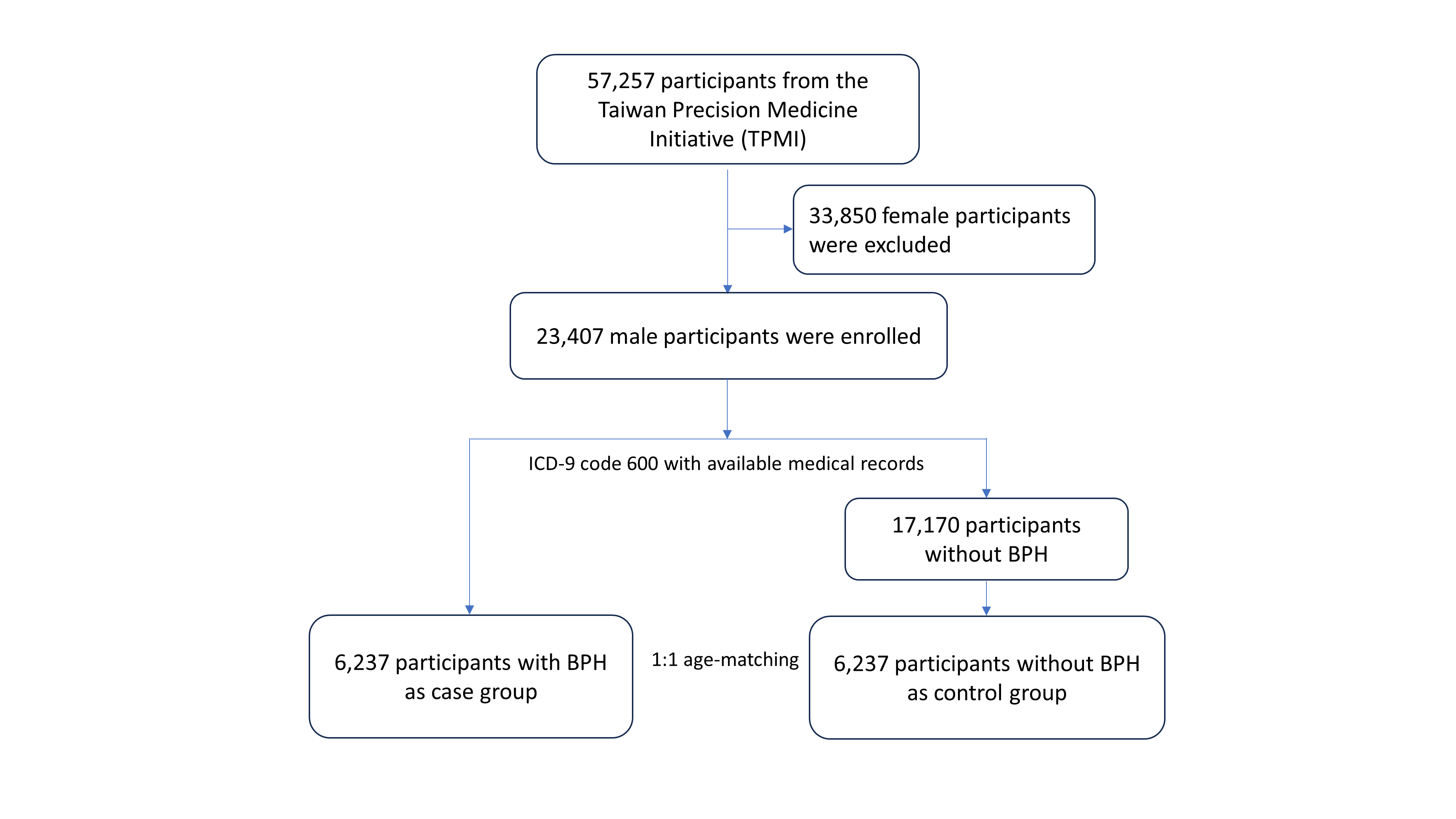

Supplement: Supplementary file 5 — Supplementary Material 5. Supplementary Fig. 3. Protein-protein association network of gene sets in PGS001865 by STRING enrichment analysis. [file 40246_2024_619_MOESM5_ESM.tif]
