# Supplementary material for: Polygenic risk score predicting susceptibility and outcome of benign prostatic hyperplasia in the Han Chinese
Source: Hum Genomics. 2024 May 22;18:49. doi: 10.1186/s40246-024-00619-3 (PMC11110300; doi:10.1186/s40246-024-00619-3)
Supplement: Supplementary file 4 — Supplementary Material 4 [file 40246_2024_619_MOESM4_ESM.docx]

| **Supplemental Table 2.** ICD-9-CM diagnosis codes. | |
| --- | --- |
| **600** | Hyperplasia of prostate |
| 600.0 | Hypertrophy (benign) of prostate |
| 600.00 | Hypertrophy (benign) of prostate without urinary obstruction and other lower urinary tract symptom |
| 600.01 | Hypertrophy (benign) of prostate with urinary obstruction and other lower urinary tract symptoms |
| 600.1 | Nodular prostate |
| 600.10 | Nodular prostate without urinary obstruction |
| 600.11 | Nodular prostate with urinary obstruction |
| 600.2 | Benign localized hyperplasia of prostate |
| 600.20 | Benign localized hyperplasia of prostate without urinary obstruction and other lower urinary tract symptoms |
| 600.21 | Benign localized hyperplasia of prostate with urinary obstruction and other lower urinary tract symptoms |
| 600.3 | Cyst of prostate |
| 600.9 | Hyperplasia of prostate unspecified |
| 600.90 | Hyperplasia of prostate, unspecified, without urinary obstruction and other lower urinary symptoms |
| 600.91 | Hyperplasia of prostate, unspecified, with urinary obstruction and other lower urinary symptoms |
|  | |

| **Supplemental Table 3.** Cases number of BPH in study population by PRS (PGS001865). | | | | | | | | | | |
| --- | --- | --- | --- | --- | --- | --- | --- | --- | --- | --- |
| **Variables** | **Total** | | **Q1** | | **Q2** | | **Q3** | | **Q4** | |
|  | **n** | **%** | **n** | **%** | **n** | **%** | **n** | **%** | **n** | **%** |
| BPH^a^ |  |  |  |  |  |  |  |  |  |  |
| No | 6237 | 50.0 | 1644 | 54.4 | 1631 | 51.7 | 1540 | 49.1 | 1422 | 45.0 |
| Yes | 6237 | 50.0 | 1377 | 45.6 | 1525 | 48.3 | 1594 | 50.9 | 1741 | 55.0 |
| ^a^ BPH = benign prostatic hyperplasia | | | | | | | | | | |

| **Supplemental Table 4.** Characteristics of the BPH study subjects (N=12,474). | | | | | | |  |
| --- | --- | --- | --- | --- | --- | --- | --- |
| Variable | | BPH case (n = 6237) | | BPH control (n = 6237) | | P-value |  |
|  |  |  |  |  |  |  |  |
| Demography | |  |  |  |  |  |  |
|  | Age | 70 ± 12 | | 69 ± 11 | | < 0.001 |  |
|  | BMI | 25.6 ± 3.8 | | 25.6 ± 3.9 | | 0.960 |  |
|  | Smoke |  |  |  |  |  |  |
|  | No | 2749 | 44.1 | 3188 | 51.1 |  |  |
|  | Yes | 3488 | 55.9 | 3049 | 48.9 | < 0.001 |  |
|  | PSA | 2.9 ± 6.8 | | 2.2 ± 12.4 | | 0.003 |  |
| Comorbidities | |  |  |  |  |  |  |
|  | Hypertension |  |  |  |  |  |  |
|  | No | 2600 | 41.7 | 3097 | 49.7 |  |  |
|  | Yes | 3637 | 58.3 | 3140 | 50.3 | < 0.001 |  |
|  | Diabetes mellitus |  |  |  |  |  |  |
|  | No | 3689 | 59.1 | 3661 | 58.7 |  |  |
|  | Yes | 2548 | 40.9 | 2576 | 41.3 | 0.623 |  |
|  | Hyperlipidemia |  |  |  |  |  |  |
|  | No | 3104 | 49.8 | 3500 | 56.1 |  |  |
|  | Yes | 3133 | 50.2 | 2737 | 43.9 | < 0.001 |  |
|  | Other cancer |  |  |  |  |  |  |
|  | No | 2993 | 48.0 | 3642 | 58.4 |  |  |
|  | Yes | 3244 | 52.0 | 2595 | 41.6 | < 0.001 |  |

^a^ Continuous variables were expressed as mean ± standard deviation (SD) and were analyzed using ANOVA follow a normal data distribution.

^b^ Categorical variables were expressed as numbers (percent) and were analyzed using the Chi-square test.

^c^ PSA, prostate specific antigen.
